# Contacts with out-of-hours primary care for nonurgent problems: patients’ beliefs or deficiencies in healthcare?

**DOI:** 10.1186/s12875-015-0376-9

**Published:** 2015-10-28

**Authors:** Ellen Keizer, Marleen Smits, Yvonne Peters, Linda Huibers, Paul Giesen, Michel Wensing

**Affiliations:** Radboud University Medical Center, Radboud Institute for Health Sciences, 114 IQ Healthcare, P.O. Box 9101, Nijmegen, 6500 HB The Netherlands; Aarhus University, Research Unit for General Practice, Aarhus, Denmark

**Keywords:** After hours care, Primary health care, Medical necessity, Motivation, Nonurgent

## Abstract

**Background:**

In the Netherlands, about half of the patient contacts with a general practitioner (GP) cooperative are nonurgent from a medical perspective. A part of these problems can wait until office hours or can be managed by the patient himself without further professional care. However, from the patient’s perspective, there may be a need to contact a physician immediately. Our objective was to determine whether contacts with out-of-hours primary care made by patients with nonurgent problems are the result of patients’ beliefs or of deficiencies in the healthcare system.

**Methods:**

We performed a survey among 2000 patients with nonurgent health problems in four GP cooperatives in the Netherlands. Two GPs independently judged the medical necessity of the contacts of all patients in this study. We examined characteristics, views and motives of patients with medically necessary contacts and those without medically necessary contacts. Descriptive statistics were used to describe the characteristics, views and reasons of the patients with medically unnecessary contacts and medically necessary contacts. Differences between these groups were tested with chi-square tests.

**Results:**

The response rate was 32.3 % (*N* = 646). Of the nonurgent contacts 30.4 % were judged as medically necessary (95 % CI 27.0-34.2). Compared to patients with nonurgent but medically necessary contacts, patients with medically unnecessary contacts were younger and were more often frequent attenders. They had longer-existing problems, lower self-assessed urgency, and more often believed GP cooperatives are intended for all help requests. Worry was the most frequently mentioned motive for contacting a GP cooperative for patients with a medically unnecessary contact (45.3 %) and a perceived need to see a GP for patients with a medically necessary contact (44.2 %). Perceived availability (5.8 %) and accessibility (8.3 %) of a patient’s own GP played a role for some patients.

**Conclusion:**

Motives for contacting a GP cooperative are mostly patient-related, but also deficiencies in access to general practice may partly explain medically unnecessary use. Efforts to change the use of GP cooperatives should focus on education of subgroups with an increased likelihood of contact for medically unnecessary problems. Improvement of access to daytime primary care may also decrease use of the GP cooperative.

## Background

In several European countries out-of-hours primary care is organised in large-scale general practitioner (GP) cooperatives [1–4]. GP cooperatives have been set up for urgent help requests that cannot wait until the regular consultation hours of the patient’s own GP. Telephone triage is used to assess the urgency of the patient’s health problem on the phone and to come to a decision about the action to be taken: refer the patient to the emergency department or ambulance care, make an appointment for a GP consultation or a home visit, provide the patient with self-care advice by telephone or advise him to visit his own GP on the next working day [2].

In the Netherlands, since the establishment of GP cooperatives in the year 2000, the number of patient contacts at GP cooperatives has increased up to 4 million contacts in 2013 (250 contacts per 1000 inhabitants per year) [5]. This increase was partly caused by patients who seek care for problems that are nonurgent from a medical perspective, leading to a disruption of the continuity of care, inefficient use of resources and avoidable high spending [6–8]. Moreover, the higher demand contributes to an increase of healthcare professionals’ workload and dissatisfaction [9]. From a medical perspective, part of the nonurgent health problems can wait until office hours or can be managed by the patient himself without further professional care. In earlier studies, GPs indicated that a substantial number of patient contacts in both primary out-of-hours care and hospital care are unnecessary and can be avoided [10, 11]. However, from the patient’s perspective, some patients perceive the need to contact a physician immediately.

There are several possible explanations for the high use of out-of-hours care for nonurgent problems. For instance, society’s experiences with expanded opening hours of other services may have led to increased expectations of healthcare availability. Also, patients want to avoid risk and perhaps expect immediate solutions for their health problems, without having to wait until the consultation hours of their own GP [12, 13]. Furthermore, families have become smaller [14] which may have resulted in parents who are less experienced with child health problems. In addition, lack of access during daytime and other deficiencies in the (primary) healthcare system may also be a motive for contacting a GP cooperative.

There have been several studies on the nonurgent and inappropriate use of the hospital emergency department (ED) [15–20], yet we know of only one study on the use of primary out-of-hours care [21]. Previous research has shown that many healthcare professionals believe health system deficiencies are an important cause of nonurgent ED use [22]. This is also indicated by patients themselves [20, 23–25] as well as policy makers. Consequently, motives for seeking ED care include lack of access to GPs (long waiting times for appointments) and dissatisfaction with the GP. These motives could also be relevant for patients visiting a GP cooperative.

Our aim was to study whether contacts with out-of-hours primary care of patients with nonurgent problems are the result of patients’ beliefs or of deficiencies in the healthcare system. We examined similarities and differences in the characteristics, views and motives of patients with medically necessary contacts and those without.

## Methods

### Design, setting, and study population

We performed a survey study in a stratified sample of 2000 patients who contacted the GP cooperative for a nonurgent health problem. The study was executed in a convenience sample of four GP cooperatives spread across the Netherlands. Two GP cooperatives took the initiative for a study on this subject themselves and the other two were selected by the researches to obtain a good variation in size and location of the participating GP cooperatives. Key features of GP cooperatives in the Netherlands have been listed in Table 1. Patients who received a triage urgency category 4 or 5 (on a scale of 0 = high to 5 = low), which was nonurgent, were included in this study. We asked the parents of patients aged under 12 to fill in the questionnaire. The following exclusion criteria were used: dying or deceased patients; patients who contacted the GP cooperative for administrative reasons or for confidential problems; patients who lived outside the Netherlands; telephone stalkers and patients who declared not to be willing to participate in our research. At one GP cooperative some high urgency patients were mistakenly included, but these were excluded based on the judgement of a GP (*N* = 21).

**Table 1:** Features of general practitioner (GP) cooperatives in the Netherlands [2, 5, 34]

Theme	Feature
General	Out-of-hours primary care has been provided by large-scale general practitioner (GP) cooperatives since the year 2000
	Participation of 50–250 GPs per cooperative with a mean of 4 hours on call per week
	About 120 GP cooperatives in the Netherlands
	Population of 100,000 to 500,000 patients with an average care consumption of 250/1000 inhabitants per year
	Out-of-hours defined as daily from 5 p.m. to 8 a.m. holidays and the entire weekend
	Patients are classified in urgency categories from high to low urgency (U1:1.5 % U2:11.1 % U3:38.0 % U4:21.7 % U5:26.3 %)
	Per shift GPs have different roles: supervising telephone triage, doing centre consultations or home visits
Location	GP cooperative usually situated in or near a hospital
	Distance of patients to GP cooperative is 30 km at most
Accessibility	Access via a single regional telephone number, meaning the first contact mostly is with a triage nurse (only 5–10 % walk in without a call in advance)
	Telephone triage by nurses supervised by GPs: contacts are divided into telephone advice (40 %), centre consult (50 %), or GP home visit (10 %)
Facilities	Home visits are supported by trained drivers in identifiable fully equipped GP cars (e.g. oxygen, intra venous drip equipment, automated external defibrillator, medication for acute treatment)
	Information and communication technology (ICT) support including electronic patient files, online connection to the GP car, and sometimes connection with the electronic medical record in the GP daily practice

At each GP cooperative 400 patients received a postal questionnaire within ten days after their contact during a four-week period between April 2009 and October 2012. Stratification was based on the type of contact: 200 questionnaires were sent to patients who had only had a telephone contact and 200 to patients who had a GP consultation at the GP cooperative. Because of a lagging response at one GP cooperative, a second group of 400 patients of that GP cooperative received the questionnaire.

### Questionnaire

The questionnaire was partly based on an existing questionnaire measuring patient satisfaction with out-of-hours care [26]. The reliability of this questionnaire was high and content and construct validity appeared to be ensured. Our questionnaire included questions about patient characteristics, views and motives.

#### Characteristics of patients

We asked the patients questions about background characteristics, duration of the problem, frequency of contact with a GP cooperative in the past year and number of contacts with their own GP for the same problem.

#### Views of patients

The questionnaire included questions about the patients’ expectations, assessment of urgency, perceptions regarding their own health, attitudes towards physical symptoms and agreement with statements on the use of the GP cooperative.

We used a shortened version of a validated questionnaire [27] for perceptions regarding their own health and attitudes towards physical symptoms. To measure perceptions regarding their own health, we used a seven-item scale which included items like “My health is worse than that of the majority of people” and “To a variety of physical symptoms, I notice something is wrong with my health”. Answers were rated on a five-point-Likert scale ranging from “totally disagree” (0) to “totally agree” (4). We summed the scores of the seven items (range 0–28) and categorised the patients into two groups: “well-perceived health” (top half: score 0–14) and “poor perceived health” (bottom half: score 15–28).

To measure attitudes towards physical symptoms, we used a five-item scale which included items like “If you do not pay attention to the signals from your body, it could be too late to detect a disease” and “If you feel something in your body, it is a sign that something is wrong”. Answers were rated on the five-point-Likert scale described above. We also summed up this score and categorised the patients in a group “not worried about physical symptoms” (top half: score 0–10) and a group “worried about physical symptoms” (bottom half: score 11–20).

#### Motives of patients

The motive categories were developed based on the literature as well as patient consultations from a previous study [28]. We used the categories: “I was worried”, “I urgently needed a GP”, “I wanted medical information”, “I needed a second opinion”, “I did not have time to go to the GP during the day”, “My own GP could not be contacted during office hours”, “I could not make an appointment on the same day with my own GP”, The ED was not prepared to help me” and “Other”.

### Medical necessity

In three rounds, two GPs independently judged the medical necessity of the contacts of all respondents. For this judgement the GPs used the information reported by the patient in the questionnaire, including age, reason for encounter, actions before and after the contact, and duration of the health care problem. A contact was scored as medically necessary if the GPs believed, based on their own medical view, that it was necessary to contact a GP during out-of-office hours. A contact was scored as medically unnecessary if the GPs believed the patient could have waited until office hours to contact their own GP or could have managed the problem with self-care. During two written consensus rounds, the two GPs discussed 159 cases (24.0 %) on which they disagreed. The Kappa was 0.40 and the proportion of the Kappa maximum was 0.45 (because the maximum attainable kappa was 0.88 we also presented the proportion of the kappa maximum [29]). After these two rounds they agreed on all cases. An example of a medically necessary contact was a 75-year-old patient presenting with acute cystitis. An example of a medically unnecessary contact was a 17-year-old patient presenting with an insect bite.

### Statistical analysis

Agreement between the two GPs on the medical necessity of the contacts was measured using percentage agreement and Cohen’s kappa.

Descriptive statistics were used to describe the characteristics, views and motives of the patients. Differences in these characteristics, views and motives between patients with medically unnecessary and medically necessary contacts were tested with chi-square tests (presenting *p*-values and degrees of freedom). Because of the large number of tests conducted, we used *p* < 0.01 to determine the significance of the differences between the two groups. We performed a nonresponse analysis at one GP cooperative for gender and age to determine whether the respondents were representative for the total study population. Data were analysed using SPSS 20 (IBM Corp. Released 2011. IBM SPSS Statistics for Windows, Armonk, NY: IBM Corp.).

### Ethics

The research ethics committee of the Radboud university medical center (CMO Arnhem-Nijmegen) stated that the study did not fall within the remit of the Medical Research Involving Human Subjects Act (WMO). Therefore, ethical approval was not needed according to Dutch law.

## Results

### Patient characteristics

The response rate was 32.3 % (*N* = 646). After exclusion of highly urgent patients (*N* = 21) 625 patients remained eligible for analysis. Of the nonurgent health care problems presented, 30.4 % were judged as medically necessary (95 % CI = 27.0-34.2). Table 2 shows background characteristics for the total group as well as the medically unnecessary and medically necessary patient contacts. Of the respondents, 59.5 % was female. Almost half of all patients contacted the GP cooperative during the weekend at daytime (47.1 %). Most of the respondents lived with a partner (73.5 %) and the largest proportion (59.0 %) lived in an urban area. About one quarter of the patients (25.2 %) had contacted their own GP at an earlier time for the same problem. More than half of the patients (57.6 %) had contact with a healthcare professional (mostly their own GP) after contact with the GP cooperative.

**Table 2:** Characteristics of patients with nonurgent problems (%)

Characteristic	Total (*N* = 625)	Medically unnecessary (*N* = 435)	Medically necessary (*N* = 190)
Gender			
Female	59.5	58.6	61.6
Age groups			
0–4	19.2	20.9	15.3
5–24	17.1	16.5	18.6
25–44	20.2	23.0*	**13.8**
45–64	23.4	21.9	27.0
≥ 65	20.0	17.7	25.4
Contact time			
Weekend daytime (8 AM–5 PM)	47.1	46.8	47.8
Evening (5 PM–11 PM)	39.8	39.8	39.7
Night (11 PM–8 AM)	13.1	13.4	12.5
Origin			
Migrant^a^	13.5	15.3	9.5
Living situation			
Cohabitation with a partner	73.5	73.7	73.3
Living environment			
Urban	59.0	60.0	56.6
Urban rural	20.8	21.3	19.6
Rural	20.3	18.7	23.8
Frequency contact GP cooperative past year			
Frequent attender^b^	26.6	29.7*	**19.4**
Duration of the problem			
Two days or more	34.0	37.9*	**25.5**
Contact with their own GP for same problem before contact with GP cooperative			
Yes	25.2	24.3	27.3
Contact with healthcare professional after contact with GP cooperative			
Yes	57.6	57.2	58.4

*p < 0.05, bold

^a^One or both parents born outside the Netherlands

^b^Contacted the GP cooperative three times or more in the preceding year

Patients with a medically unnecessary contact were significantly more often between the ages of 25–44 years (23.0 % versus 13.8 %; *χ*^2^ = 7.06, df = 1, *p* = 0.004) compared to patients with a medically necessary contact. They were also more often frequent attenders (29.7 % versus 19.4 %; *χ*^*2*^ = 6.17, df = 1, *p* = 0.007) than patients with a medically necessary contact. Of the patients with a medically unnecessary contact, 37.9 % had contacted the GP cooperative with a problem that had existed for several days, which was a significantly higher number than patients with a medically necessary contact (25.5 %; *χ*^*2*^ = 8.90, df = 1, *p* = 0.002). Although not statistically significant, patients with a medically unnecessary contact were more often migrants than patients with a medically necessary contact (15.3 % versus 9.5 %).

### Patient views

Table 3 shows the views of the patients. Patients with a medically unnecessary contact expected to see a doctor less often than patients with a medically necessary contact (66.7 % versus 78.1 %; *χ*^*2*^ = 7.99, df = 1, *p* = 0.003), more often thought their problem was nonurgent (27.5 % versus 16.6 %; *χ*^*2*^ = 8.00, df = 1, *p* = 0.003) and more often believed that the GP cooperative is intended for all help requests (51.4 % versus 36.4 %; *χ*^*2*^ = 5.77, df = 1, *p* = 0.008). There were no differences in judgement of one’s own health (13.5 % versus 10.9 %) and attitude towards physical symptoms (23.1 % versus 20.9 %) between the two groups.

**Table 3:** Views of patients with nonurgent problems (%)

View	Total (*N* = 625)	Medically unnecessary (*N* = 435)	Medically necessary (*N* = 190)
Expectation			
Expecting to see a doctor	70.1	66.7*	**78.1**
Perception of urgency			
Nonurgent	24.2	27.5*	**16.6**
Perception of patient’s own health			
Poor	12.7	13.5	10.9
Attitude towards physical symptoms			
Worried	22.4	23.1	20.9
GP cooperative is intended for all requests for help^a^			
Agree	46.6	51.4*	**36.4**

*p < 0.05, in bold

^a^Question asked in two GP cooperatives

### Motives for contacting the GP cooperative

Patients with nonurgent health problems most frequently mentioned patient-related motives for contacting the GP cooperative (Table 4). The most frequently mentioned motive for patients with a medically unnecessary contact was worry about their own health (45.3 %; medically necessary 33.2 %; *χ*^*2*^ = 7.81, df = 1, *p* = 0.005). In contrast, the most important motive for patients with a medically necessary contact was a perceived need for urgent contact with a GP (44.2 %; medically unnecessary 29.8 %; *χ*^*2*^ = 13.27, df = 1, *p* = 0.000). Furthermore, patients with a medically unnecessary contact more often reported a need for medical information as opposed to patients with a medically necessary contact (29.3 % versus 16.8 %; *χ*^*2*^ = 10.55; df = 1, *p* = 0.001).

**Table 4:** Motives of patients with nonurgent problems for contacting a GP cooperative^a^ (%)

Motive	Total (*N* = 625)	Medically unnecessary (*N* = 435)	Medically necessary (*N* = 190)
Patient-related motives			
I was worried	41.6	45.3*	**33.2**
I urgently needed a GP	34.3	29.8*	**44.2**
I wanted medical information	25.5	29.3*	**16.8**
I needed a second opinion	1.6	2.1	0.5
I did not have time to go to the GP during the day	1.5	1.4	1.6
Healthcare system-related motives			
My own GP could not be contacted during office hours	8.3	10.1	4.2
I could not make an appointment on the same day with my own GP	5.8	4.5	8.9
The ED was not prepared to help me	1.6	1.2	2.6
Other	14.1	12.7	17.4

^a^Multiple answers were possible

*p < 0.05, in bold

Healthcare system-related motives, such as telephone accessibility of daytime general practices and availability of the patient’s own GP for appointments, were less frequently mentioned. Of the patients with medically unnecessary contacts 10.1 % indicated that their own GP could not be contacted during office hours (medically necessary 4.2 %). Of the patients with medically necessary contacts 8.9 % indicated that they could not make an appointment on the same day with their own GP (medically unnecessary 4.5 %).

## Discussion and conclusion

### Discussion

#### Main findings

Nearly two thirds of the nonurgent patient contacts were medically unnecessary, while the majority of these patients assessed their problems as urgent. Patients with medically unnecessary contacts differ from patients with necessary contacts in several ways. They are younger, they are more often frequent attenders and they more often have a problem that had already existed for several days. They also assess their own problem more often as nonurgent, expect to see a doctor less often, and more often think that the GP cooperative is intended for all help requests as opposed to the group with medically necessary contacts. The groups do not differ in their perception of their own health and physical symptoms. Furthermore, we found that patients with medically unnecessary contacts appeared to be more often migrants (not statistically significant).

Patient-related motives, such as worry, a perceived need to see a GP and a need for medical information were the most important motives for contacting a GP cooperative for all patients. Worry was the most frequently mentioned motive for patients with a medically unnecessary contact, while a perceived need for urgent contact with a GP was the most often mentioned motive for patients with a medically necessary contact. Healthcare system-related motives, such as deficiencies in availability and accessibility of a patient’s own GP, were also mentioned by some patients.

#### Comparison with existing literature

Previous studies at the ED also showed that patients with medically unnecessary contacts were younger and were more often migrants [17, 30, 31]. A Canadian study found that 33 % of the patients who visited a walk-in clinic felt their symptoms were of low urgency. This is somewhat higher than we found (24 %) [32].

The most important motives for contacting a GP cooperative we found were in accordance with the study of Shipman et al., who found that concern, anxiety and the need for advice, explanation and reassurance were motives for contacting out-of-hours services [21]. For 10 % of the patients with medically unnecessary contacts the inaccessibility of their daytime GP practice was a motive for contacting the GP cooperative. Other studies showed the same percentage (10 %) [33] or higher (21 %) [12], but the study population differed from ours as there was no selection of nonurgent contacts. In addition, other studies found a relation between poorer telephone accessibility in daytime primary care and a higher consumption at the GP cooperative [34, 35]. The results of this study may also be representative for other countries with a well-developed primary care system. In this type of system, the general practice is the usual point of entry to healthcare and the GP has a coordinating role in the delivery of healthcare.

#### Strengths and Limitations

This is, as far as we know, the first study on motives and views from patients with nonurgent health problems who contact the GP cooperative, comparing medically necessary and unnecessary nonurgent contacts. Not all nonurgent contacts are by definition medically unnecessary and thus inappropriate. For that reason, we have focused on the patients who did not need professional care out-of-hours from a medical perspective.

Our study covers a relatively large group of patients, although a limitation of the study is the relatively low response rate. A systematic review showed that other patient satisfaction questionnaires in the setting of out-of-hours primary care services had higher response rates (39.7 % to 45.7 %) [36]. Therefore, it is difficult to determine whether our results can be generalised to the whole patient population. In a non-response analysis at one GP cooperative we found that responders seemed slightly older than non-responders. Additional analysis showed that patients in older age groups had more medically necessary contacts, so the group of patients with medically unnecessary contacts could be larger in reality. There was almost no variation in answers between these four GP cooperatives: the self-selected GP cooperatives did not differ from the GP cooperative selected by the researchers. Moreover, the GP cooperatives were spread across the Netherlands, thereby contributing to the representativeness of the results for our country.

The medical necessity of the patients’ contacts was judged by two GPs based on information given by the patients in the questionnaire. The medical records of the patients were not available for this study, due to the confidentiality of such information. However, the GPs indicated that they had enough information on all cases to make a good judgement on the medical necessity of the contacts.

### Practice implications

Most patients with a medically unnecessary contact believe their health problem is urgent, thus justifying their contact with the GP cooperative. Yet, there is also a group who assesses their problem as nonurgent. They do not seem to be insecure about their own health and physical symptoms. In order to reduce the number of medically unnecessary patient contacts, patients should be informed of the purpose of the GP cooperative: it is intended for urgent problems that cannot wait until the next day. Frequent attenders especially, patients between 25 and 44 years old and migrants should be informed. This can be done by the GP and the triage nurse at the GP cooperative, but also by their own GP who will be informed the day after a patient contacted a GP cooperative. In addition, GPs could provide more self-care advice about nonurgent illnesses during consultations and encourage the use of internet information, because a substantial group of patients contacts the GP cooperative for medical information. This will possibly prevent patients from contacting the GP cooperative with similar health problems in the future.

The above recommendations focus on changing patient behaviour, which could prove to be a difficult aspect to influence. Other ways of reducing medically unnecessary contacts can be found in healthcare system adjustments. Although only a small group of patients with a medically unnecessary contact mentioned accessibility as a motive for contacting out-of-hours care, improvement of access to their own GP during the day may optimise use of the GP cooperative. This could be accomplished by optimising telephone accessibility during the day and possibilities for same-day appointments [34, 35].

### Conclusion

Motives for contacting a GP cooperative are mostly patient-related, but also deficiencies in access to general practices may partly explain medically unnecessary use. Efforts to change the use of GP cooperatives should focus on education of subgroups with an increased risk of contact for medically unnecessary problems. Improvement of access to daytime primary care may also decrease use of the GP cooperative.

## Abbreviations

GP: General Practitioner
ED: Emergency Department
CI: Confidence Interval
